# Advances in Biosynthesis, Regulation, and Function of Apple Cuticular Wax

**DOI:** 10.3389/fpls.2020.01165

**Published:** 2020-08-05

**Authors:** Ya-Li Zhang, Chun-Xiang You, Yuan-Yuan Li, Yu-Jin Hao

**Affiliations:** State Key Laboratory of Crop Biology, Shandong Collaborative Innovation Center of Fruit & Vegetable Quality and Efficient Production, College of Horticulture Science and Engineering, Shandong Agricultural University, Tai-An, China

**Keywords:** composition, structure, function, regulation, cuticular wax

## Abstract

A layer of cuticular wax is deposited on the surface of terrestrial plants, which reduces the damage caused by environmental stress and maintains growth in a relatively stable internal environment. Apple cuticular wax is an important part of the fruit epidermis that plays an essential role in apple development, storage, and adaptation to environmental stress. The formation of cuticular wax has been described at the transcriptional, post-transcriptional, and translational levels in Arabidopsis, whereas less research has been performed on apple cuticular wax. Here, we provide a brief overview of how apple cuticular wax is formed, as well as its structure, composition, and function. An association among the environment, genes, and apple cuticular wax deposition was revealed. Cuticular wax prevents fruit rust from occurring on apple. Taken together, a detailed understanding of apple cuticular wax is discussed. The results will act as a reference for extending the storage period and increasing the commodity value of apple.

## Introduction

Plant growth is a process of continuous adaptation to the environment (Kim et al., 2019; Trivedi et al., 2019). About 45 billion years ago, aquatic plants began to evolve towards land to adapt to the changing environment. Terrestrial plants formed a hydrophobic cuticle on the surface of their aerial organs to protect themselves from water loss (Waters, 2003; Leliaert et al., 2011; Sørensen et al., 2011; Budke et al., 2012). The interactions between plants and their environment are vital for the life of plants under changing environmental conditions. One such interaction is the emergence of the cuticle on aerial organs of land plants (Bargel et al., 2006). Apple is a common fruit. The primary role of apple cuticular wax is to reduce non-stomatal water loss and prevent pathogenic attacks. The cuticular wax of apple directly determines its appearance quality and market value. In this study, we summarize the biosynthesis, composition, regulation, and function of apple cuticular wax to provide deeper knowledge of apple cuticular wax and to provide a basis for studying the molecular mechanism of apple cuticular wax biosynthesis.

## Crystal Morphology and Composition of Apple Cuticular Wax

The epidermal wax, located in the outermost layer of apple cuticular wax, is in direct contact with the external environmental (Kunst and Samuels, 2003) and is one of main components of the cuticle responsible for epidermal permeability (Veraverbeke et al., 2001). Epidermal wax generally presents with different crystal morphology from other apple cuticular wax under a scanning electron microscope. The epidermal wax of apple often has a platelet structure, and this difference is significant among different developmental stages and apple varieties (Curry, 2005). Many irregular lamellar crystals are detectable on the surface of “Granny Smith” and “First Red”; however, the epidermis of “Red Rome” is smooth, with a small number of parallel wax crystals (Belding et al., 1998). The results of chemical composition are consistent with the crystalline structure (Chu et al., 2017). Earlier studies showed that the crystal morphology of apple cuticular wax is more susceptible to environmental influences compared to its composition (Belding et al., 2000). The environmental conditions of light, temperature, and humidity affect the crystal morphology of apple epidermal wax (Roy et al., 1994; Li F. et al., 2019; Zhang et al., 2019b). The bagging technology during apple production changes the microenvironment around the apples, which affects the wax crystal morphology of the epidermis, making the fruit surface smooth.

The components of apple cuticular wax are long-chain alkanes, alcohols, aldehydes, fatty acids, and ketones, which are aliphatic VLCFAs. In addition, apple cuticular wax contains triterpenes, which are a unique component of the fleshy fruit epidermis (Belding et al., 1998; Trivedi et al., 2019). Triterpenes and alkanes are the main components of apple cuticular wax (Belding et al., 2000). The content of these main ingredients fluctuates due to the effects of species and environmental conditions. Alkanes, primarily C29 alkanes, comprise 16.6–49% of apple cuticular wax contents. Primary and secondary alcohols occupy 0–20.4% of total apple cuticular wax. Aldehydes, fatty acids, and ketones comprise only a small portion of total apple cuticular wax (0–6.0%), and the percentage of triterpenes of total wax content varies from 32 to 70% (Curry, 2008; Chu et al., 2017). Different apple surface features have different epidermal wax compositions. The glossy quality of apples is largely due to the alcohol in the epidermal wax (Veraverbeke et al., 2001). The greasy surface characteristics of apples during storage are mainly due to fluctuations in secondary alcohols (Veraverbeke et al., 2001; Yang et al., 2017). One of the wax components in apple epidermis, alpha-farnesene, leads to the accumulation of superficial scald on apples (Rowan et al., 2001). Alkane content decreases and fatty acid content increases during storage of apples (Dong et al., 2012).

## Biosynthesis, Regulation and Deposition of Apple Cuticular Wax

### Biosynthesis

The cuticular wax biosynthetic pathways have been reported in Arabidopsis (Lee and Suh, 2013; Yeats and Rose, 2013), but they remain unclear in apple. Apple cuticular wax contains the same composition as that of Arabidopsis, including alkanes, alcohols, aldehydes, fatty acids, wax ester, and ketones (Belding et al., 1998), we speculate that the same wax components have similar synthetic pathways. Furthermore, apple evolved a unique synthetic pathway for triterpenes. Identifying the wax synthesis-related genes is conducive to our objective (Lashbrooke et al., 2015; Qi et al., 2019; Zhang et al., 2019a; Zhang et al., 2019b; Zhong et al., 2020). The apple expression sequence tag and genomic sequence analyses identified candidate genes, including *CER1*, *CER4*, *CER10*, *LACS2*, *KCS7/2*, *LCR*, *FDH*, *PAS2*, *WBC11*, *LTPG1*, and *WIN1*, which are specifically expressed in the peel of different apple varieties. These studies suggest that these genes may participate in the synthesis of apple skin wax (Velasco et al., 2010; Albert et al., 2013). Therefore, we speculated that the apple synthetic pathways are partly similar to those in Arabidopsis. Very long chain fatty acids (VLCFAs) are precursors of cuticular wax biosynthesis, and its derivatives that subsequently form are the main ingredients of plant cuticular wax (Bernard and Joubès, 2013). Epidermal wax synthesis is the first *de novo* synthesis of C16 and C18 fatty acids that is activated into C16- and C18-coenzyme A (CoA) by long chain acyl–CoA synthetase (Yeats and Rose, 2013). VLCFAs form alcohols and wax esters through the alcohol-forming pathway, and aldehydes, alkanes, secondary alcohols, and ketones are produced through the alkane-forming pathway (Moggia et al., 2016; Yang et al., 2017).

**Figure 1:**
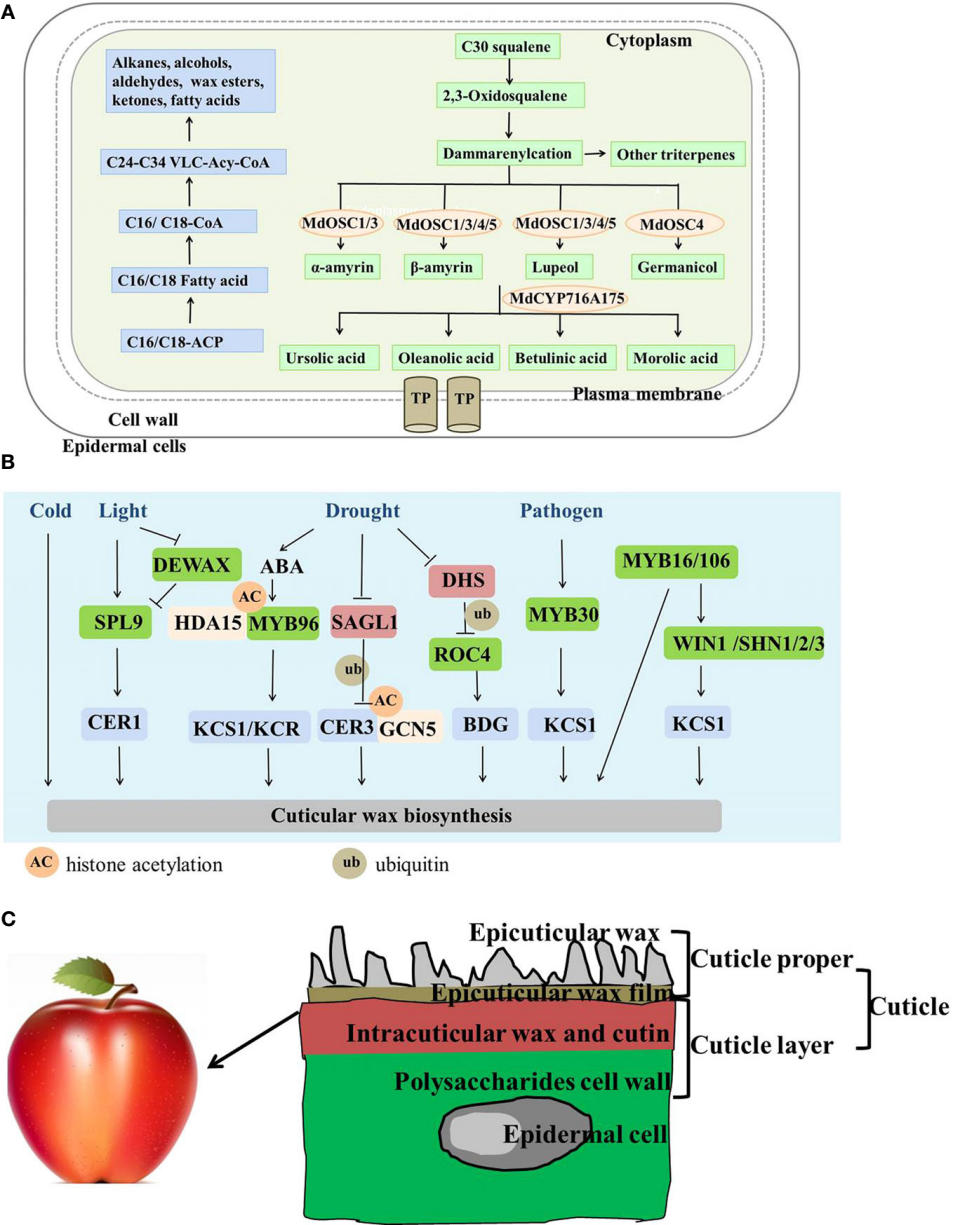
A model of the biosynthesis, regulation, and deposition of apple cuticular wax based on progress made in apple and other plants. **(A)** Apple cuticular wax biosynthetic pathway. Blue and green represent the same and different synthetic pathways as in Arabidopsis. TP, transport protein. **(B)** Environmental and genetic co-regulatory networks of cuticular wax biosynthesis at different regulatory levels. Blue represents the structural genes related to cuticular wax synthesis; green represents the transcriptional factors related to cuticular wax synthesis; red represents E3 ubiquitin ligase; orange represents the histone acetylation genes (HDA15 and GCN5). **(C)** Simple deposition diagram of the apple fruit cuticle. Different colors represent different apple cuticular wax components and positions. The graphics are not drawn according to the true ratio.

The precursors and pathways of triterpene biosynthesis have been revealed in apple (**Figure 1A**). Pentacyclic triterpenes account for large proportion of apple epicuticular waxes (Belding et al., 1998). Along with long chain fatty acids and secondary metabolites, triterpene biosynthesis occurs particularly in the waxy coating of leaves and fruits, such as apples and pears (Kunst and Samuels, 2003). Ursolic acid (UA), oleanolic acid (OA), and betulinic acid (BA) are the main triterpene types in most commercial apple varieties (McGhie et al., 2011). Triterpenoids are synthesized from the 30-carbon intermediate squalene, and squalene is converted into 2,3-oxidosqualene by squalene epoxidase (Brendolise et al., 2011). The first step in the biosynthesis of all triterpenes is cyclization of the 30-carbon precursor 2,3-oxidosqualene. Lupeol, α-amyrin, β-amyrin, and germanicol are the primary carbon framework of apple triterpenes. They are cyclized by members of the oxidosqualene cyclase (OSC) family (Phillips et al., 2006). MdOSC1 and MdOSC3 encode a multifunctional oxidosqualene cyclase that produces α-amyrin, β-amyrin, and lupeol. MdOSC4 and MdOSC5 cyclize 2,3-oxidosqualene into lupeol and β-amyrin. In addition, MdOSC4 cyclizes the production of germanicol. Finally, MdCYP716A175 catalyzes the C-28 oxidation of α-amyrin, β-amyrin, lupeol, and germanicol, producing UA, OA, BA, and morolic acid (Andre et al., 2016).

### Regulation

Cuticular wax biosynthesis and deposition are co-regulated by environmental factors and genetic characteristics (Hen-Avivi et al., 2013; Lee and Suh, 2013). Environmental stimuli include: humidity, light, temperature, and pathogen load. As for humidity, drought conditions make the epidermal wax more complete, preventing water loss to ensure a normal supply of water for apple (Trivedi et al., 2019). Studies in various species have shown that more cuticular wax is deposited under light than dark conditions (Li R. J. et al., 2019). In addition, the morphology and properties of apple cuticular wax change directly with temperature (Roy et al., 1994). As apple cuticular wax is closely associated with post-harvest storage, the temperature is critical for post-harvest storage (Lara et al., 2014). Formation of a thicker cuticular wax is one of the strategies plants use to resist pathogen infection. In general, an increase in deposition of cuticular wax has been detected in apple infected with a pathogen (Zhang et al., 2019b).

The deposition of cuticular wax has been regulated at the transcriptional, post-transcriptional, and translational levels in apple and other model plants. Structural genes that encode enzymes have strong effects on cuticular wax. Apple MdCER1 and MdCER2 affect cuticular wax permeability and resistance to drought by promoting the formation of epidermal wax (Qi et al., 2019; Zhong et al., 2020). Cuticular wax is also regulated at the transcriptional level. SPL9 positively regulates ECERIFERUM1 (CER1) to significantly alter cuticular wax contents in response to light. DEWAX forms a heterodimer with SPL9 and interferes with SPL9 DNA binding ability to CER1, revealing how changes in the light/dark cycle alter epidermal wax deposition (Li R. J. et al., 2019). MYB16 and MYB106 coordinate with WIN1/SHN1 to regulate cutin and VLCFA biosynthesis. MYB106 induces the expression of *WIN1/SHN1*, which is involved in the regulatory cascade of cuticle development (Aharoni et al., 2004; Oshima et al., 2013; Zhang et al., 2019a). MYB96 promotes cuticular wax biosynthesis by directly binding to the KCS/KCR promoters in response to abscisic acid (ABA)-mediated drought (Seo et al., 2011). Additionally, *MYB30* expression is induced by infection of bacterial pathogens, leading to upregulated expression of the *FAE* complex; thus, positively regulating epidermal wax deposition (Raffaele et al., 2008; Zhang et al., 2019b). Improper cuticular wax deposition is accompanied by decreased expression of the wax biosynthetic genes and MdSHN3 TF in rusty apples (Lashbrooke et al., 2015; Legay et al., 2015). In addition, cuticular wax biosynthesis is regulated at the translational and post-translational levels. For example, GCN5 regulates H3K9/14 acetylation at the *CER3* promoter regions involved in the accumulation of stem cuticular wax, which emphasizes the epigenetic involvement in cuticular wax biosynthesis (Wang T. et al., 2018). MYB96 positively regulates cuticular wax deposition, as it recruits the histone modifier HDA15 to participate in ABA signaling (Seo et al., 2011; Lee and Seo, 2019). A (RING)-type protein DROUGHT HYPERSENSITIVE (DHS) mediates ubiquitination of ROC4, and weakens its binding to downstream structural genes to regulate wax synthesis in response to drought conditions (Wang Z. et al., 2018). The F-box E3 ubiquitin linkages SMALL AND GLOSSY LEAVES1 (SAGL1) mediates proteasome-dependent degradation of CER3 in response to changes in humidity (Kim et al., 2019). A regulatory network about how plants alter wax content to cope with environmental change is depicted based on previous studies (**Figure 1B**). These results will play an important role in the study of apple wax synthesis.

### Deposition

The main ingredient of cuticular wax is synthesized in the endoplasmic reticulum, but deposited on the plant surface (Kunst and Samuels, 2003). Increasing evidence suggests that the LTP and ABC proteins play an important role in the transfer and deposition of monomers during cutin self-assembly in Arabidopsis (McFarlane et al., 2010; Kim et al., 2012). It is unclear whether these two transport proteins and/or others are involved in the transport of apple epidermal wax, which needs to be demonstrated by subsequent studies.

After transport, the cuticle is deposited in the aerial organs of land plants, including the stems, leaves, flowers, and fruits (Pollard et al., 2008). The deposition and distribution of cutin can be observed by transmission electron microscopy. Cutin forms a sealed protective layer around the surface of the apple to prevent non-stomatal water loss and invasion by pathogenic bacteria (Martin and Rose, 2013). Deposition of the wax layer is generally accompanied by cutin as when the secondary cell wall is lignified (Yeats and Rose, 2013). The deposition structure of the apple fruit cuticle is described in **Figure 1C**. The cuticle is primarily composed of cutin/cutan, which are polymerized from *W*-hydroxy fatty acids and waxes synthesized from very-long-chain aliphatic molecules (Lara et al., 2014). The epidermal wax film and epidermal wax crystals cover the cuticle proper, which is a mixture of intracuticular wax and cutin. The cuticular layer mainly includes cell wall polysaccharides and cutin and may also contain intracuticular waxes (Yeats and Rose, 2013). The cuticle proper and the cuticle layer constitute the cuticle.

## Function of Apple Cuticular Wax

As apple cuticular wax accompanied the evolution of land plants, cuticular wax is the indemnification of the survival of plants in a new terrestrial environment. Similarly, the role of cuticular wax in apple has been demonstrated. One of the functions of apple cuticular wax is to prevent water loss. The cuticle plays a major role as a barrier for water and solutes and regulates gas exchange when stomata are closed or are not present (Riederer and Schreiber, 2001). Aquatic and terrestrial plants have different abilities to exchange CO_2_ and O_2_ due to differences in cuticular wax (Kim et al., 2019; Trivedi et al., 2019). Apple varieties with thicker cuticular wax suffer from less water loss and can be stored for a longer period compared to those with thinner wax layers (Knoche and Grimm, 2008; Curry, 2012).

In addition, the cuticle protects the plant against pathogenic attack. Forming a thick wax layer on the apple epidermis is a strategy to resist pathogen infection (Veraverbeke et al., 2001). The wax layer prevents infection of pathogens because epicuticular wax self-cleans, so dust or bacteria are readily removed from the plant surface (Bargel et al., 2006). Another possible reason is creating conditions that are not beneficial for the majority of plant pathogens. Pathogen infection and reproduction generally require humid conditions, but the water-repellency of epicuticular waxes has an extreme water removal capacity and hence the surface is virtually dry, which significantly controls the growth of pathogenic bacteria (Li-Beisson et al., 2013). A disrupted wax microstructure or environmental pollutants can cause a significant increase in fungal spores during the development of apple fruit or during postharvest storage (Samuels et al., 2008).

Fruit rust is a common disease of apple cultivars, which adversely affects the appearance of fruits (Faust and Shear, 1972). Economic losses are caused by fruit rust because consumers prefer apples with a waxy-skin without rust. Microscopic cracks in the cuticle cause a disorder of the fruit skin known as russet (Wood, 2001). Apple russet results from the appearance of micro-cracks and the formation of a corky suberized layer (Lashbrooke et al., 2015). Severe destruction of the waxy skin is prerequisite for the formation of apple rust. The arrangement of the epidermal cells and the thickness of the cuticular wax on the fruit surface are essential factors affecting the formation of apple rust (Curry, 2012; Legay et al., 2015). The balanced distribution of wax, cutin, suberin, and lignin is beneficial to keep the apple surface glossy and clean (Wang et al., 2020). Intact cuticular wax is indispensable to extend the shelf life of apples. Damage to the integrity and order of the epidermal wax can lead to the formation of rust or increase the potential for the occurrence of rust. Fruit rust has a strong relationship with the surface structure of the fruit (Khanal et al., 2013). Non-rust apple varieties generally have the characteristics: neatly arranged cells, a uniform wax layer, a tight stratum corneum layer, and few gaps (Knoche and Grimm, 2008; Legay et al., 2015). Studies have shown that the composition of triterpenes is closely related to apple rust. Differences in terpenoid components may be the cause of fruit rust. UA and OA are significantly predominant in waxy apple, whereas BA significantly dominates in russeted apples (Andre et al., 2016). Triterpene-caffeates have been detected in suberized tissues, such as russeted apple skin and apple bark, but not in waxy, nonsuberized apple skin (Brendolise et al., 2011; Andre, 2013). In addition to being related to apple rust, triterpenes also display a wide range of important biomedical properties, including anti-inflammatory, anti-cancer (He and Liu, 2007), anti-HIV, and antifungal (Andre et al., 2012; Szakiel et al., 2012). New elite apple varieties could be developed using apples rich in triterpenes. These apple triterpenes can be used in the medical industry after genetic engineering.

## Prospects

Apple cuticular wax balances the distribution of nutrients on the apple surface, resists mechanical stress and pathogen infection, maintains physiological integrity, and prolongs the fruit storage period. Until now, the main components, crystalline structure, and metabolic pathways of apple cuticular wax were clear, several structural genes and transcription factors have also been identified to be involved in apple cuticular wax regulatory pathways. However, the regulatory pathways and networks at the molecular level remain largely unknown, which need to be investigated in the future. In addition to its features as a protective barrier, apple cuticular wax directly determines the glossy quality of apples. However, which components and crystalline structures are crucial for glossy quality are unknown. Also, the research methods and techniques to determine fruit glossy quality are difficult. There is not a complete system established to study apple glossy quality; therefore, new technologies and methods need to be developed to determine apple glossy quality.

## Author Contributions 

Y-YL and Y-JH initiated and designed the overall concept. Y-LZ wrote the manuscript. Y-YL, Y-LZ and C-XY revised the manuscript. All authors approved the final version and approved it for publication.

## Funding

This work is supported by the National Key R&D Program of China (2018YFD1000200), the National Natural Science Foundation of China (31772275), and the Natural Science Fund for Excellent Young Scholars of Shandong Province (ZR2018JL014).

## Conflict of Interest

The authors declare that the research was conducted in the absence of any commercial or financial relationships that could be construed as a potential conflict of interest.
